# Comparison on Virulence and Immunogenicity of Two Recombinant Vaccinia Vaccines, Tian Tan and Guang9 Strains, Expressing the HIV-1 Envelope Gene

**DOI:** 10.1371/journal.pone.0048343

**Published:** 2012-11-06

**Authors:** Rong Zhu, Weijin Huang, Wenbo Wang, Qiang Liu, Jianhui Nie, Shufang Meng, Yongxin Yu, Youchun Wang

**Affiliations:** 1 Department of Cell Biology, Key Laboratory of the Ministry of Health for Research on Quality and Standardization of Biotech Products, National Institutes for Food and Drug Control, Beijing, China; 2 The First Department of Viral Vaccine, Key Laboratory of the Ministry of Health for Research on Quality and Standardization of Biotech Products, National Institutes for Food and Drug Control, Beijing, China; 3 Wuhan Institute of Biological Products, Wuhan, China; University of Rochester, United States of America

## Abstract

**Background:**

The vaccinia virus Guang9 strain (VG9), derived from the vaccinia virus Tian Tan strain (VTT) has been found to be less virulent than VTT.

**Methodology/Principal Findings:**

To investigate whether VG9 could be a potential replicating virus vector, the *TK* genes in VG9 and VTT were replaced with the HIV-1 envelope gene via homologous recombination, resulting in the recombinant viruses, VG9-E and VTT-E. The biology, virulence, humoral and cellular immunological responses of VG9-E and VTT-E were evaluated. Our results indicated no obvious difference in range of host cells and diffusion between two recombinant viruses. Neurovirulence for VG9-E in weanling and suckling mice, and skin virulence in rabbits, were lower than that of VTT-E. The humoral immune responses, including binding antibody and neutralizing antibody responses, induced by VG9-E were not significantly different from those for VTT-E whilst IFN-γ response which represented cellular immune response induced by VG9-E was significantly higher than that did by VTT-E.

**Conclusions/Significance:**

Our results indicated that VG9-E was less virulent, yet induced higher cellular immune response than VTT-E. Therefore, it could be an ideal replicating vaccinia vector for HIV vaccine research and development.

## Introduction

The vaccinia virus Tian Tan strain (VTT) has been used as a vaccine against smallpox and played a vital role in the eradication of smallpox in China. Serious adverse side-effects, such as gangrene and post-vaccination encephalitis, have been reported in a few cases among millions of people inoculated with VTT. This was possibly because it retained a level of neurovirulence, despite of the fact that an attenuated strain was used [1]. To obtain a safer and more effective attenuated strain of vaccinia virus, vaccinia virus Guang9 strain (VG9) was isolated by successive plaque-cloning purification from VTT in 1970 [2]. This strain resulted in a lower pock diameter, less swelling, smaller necrosis area, and lower incidences of fever and hyperpyrexia [2]–[4]. The virulence of VG9 in various animal models was found to be lower than its parental virus, VTT; however, it was still neurotoxic to some extent. The VG9 strain was 100-fold less virulent than VTT in weanling mice and 18-fold less virulent in suckling mice. With respect to virulence in rabbits, when infected by intradermal inoculation, the duration of red swelling on the skin was shorter with a rapid recovery. Only slight necrosis was induced with a higher virus titer (10^7.54^ PFU) for VG9, compared with severe necrosis that developed using a lower titer (10^6.63^ PFU) of VTT. The mean necrotic diameter induced by 10^5.63^ PFU of VTT was almost the same as that induced by 10^7.54^ PFU of VG9 [5].

Peng *et al.* reported that using a replication-competent adenovirus as a vector produced better protection than replication-deficient virus against SIV challenge. This indicated that the replicating virus vector had the advantage of inducing a stronger immune response to a target protein than a non-replicating virus vector [6]. Thus, developing other replicating vectors, such as poxvirus, to defeat pathogens has been encouraged [7]. However, most replicating viral vectors may induce adverse reactions in humans. Therefore, it is important to develop a replicating vector with high immunogenicity but low virulence. VG9 was isolated from VTT in our laboratory and its virulence was lower than that observed in the parental strain. To investigate whether VG9 is a potential candidate of replicating vector, recombinant VG9 and VTT were constructed incorporating the HIV-1 envelope protein (*env*) gene. Their virulence, along with humoral and cellular immunologic responses were evaluated and compared.

## Results

### Construction of pDRVISV1.0-env, VG9-E and VTT-E

The DNA vaccine, pDRVISV1.0-*env*, containing HIV-1 *env* fragment was confirmed by specific endonuclease digestion and sequencing. The recombinant shuttle vector, pJSC1175-*env* containing the HIV-1 *env* fragment was constructed and confirmed using specific endonuclease digestion and sequencing of the PCR amplicon. Following homologous recombinant between the recombinant shuttle vector and VTT or VG9, the two recombinant vaccinia viruses (VTT-E and VG9-E) containing HIV-1 *env* were confirmed by PCR, western blot analysis and immunofluorescence.

### Viral Replication and Immunostaining

**Figure 1:**
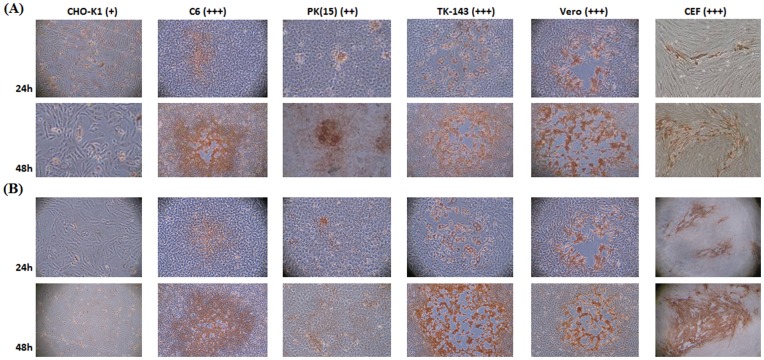
Infection and cell-to-cell spread of VG9-E and VTT-E in six cell types. Confluent cells were infected at an MOI of 0.05 with VG9-E (A) and VTT-E (B), fixed at 24 and 48 h post-infection, respectively, then incubated with an anti-VV-specific polyclonal antibody and immunostained at 24 and 48 h pi.

The two recombinant viruses were able to infect six different cell lines [C6, CHO-K1, PK (15), TK-143, Vero and CEF] (Fig. 1), and could diffuse in all cell lines, except for CHO-K1. The host cells for VG9-E and VTT-E were the same as their parental strains (data not shown) as determined by immunostaining [8]. The cytopathic effect (CPE) and plaques in permissive cells infected with VG9-E were evidently later than those infected with VG9. The replication and spread of both VG9-E and VTT-E were indistinguishable from that observed for VG9 and VTT in these cells.

### ICLD_50_

To investigate the neurovirulence *in vivo* of VG9-E and VTT-E, BALB/c and suckling mice were inoculated via the intracranial (i.c.) route [9]–[11]. All tested BALB/c mice grew normally and no apparent symptoms were observed during the 14 days after VG9-E inoculation (10^6.54^ PFU). One of the BALB/c mice died after i.c. injection with VTT-E (10^6.54^ PFU). In suckling mice, the ICLD_50_ for VG9-E was 15 times lower than that of VTT-E(10^5.86^ PFU vs 10^4.69^ PFU).

### Skin Virulence in Rabbits

**Figure 2:**
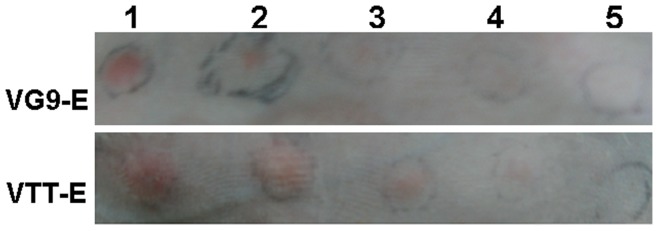
Skin virulence of VG9-E and VTT-E in rabbits at day 5 post-inoculation. 10^7.06^ *(lane1)*, 10^6.06^ *(lane2)*, 10^5.06^ *(lane3)*, 10^4.06^ *(lane4)* PFU VG9-E and VTT-E and the same volume of PBS *(lane5)* were injected intradermally on one side of each animal’s dorsal spine. The red swelling and necrosis sizes were observed.

After four rabbits were inoculated with diluted virus stocks of VG9-E and VTT-E, the general inflammation reaction for both recombinant viruses was observable at some inoculation sites on the third day and reached a maximum on the fifth day, but no skin necrosis was observed. The duration of the inflammation reaction was short, with the size of the inflamed area reducing from day 6, and disappearing completely within 10 days. As shown in table 1 and Fig. 2, red swelling size at 10^6.06^ PFU VG9-E injecting site was the same as that of 10^4.06^ PFU VTT-E injecting site on 4^th^ and 5^th^ days post injection. Our results indicated that the skin virulence of VG9-E in rabbits was much lower than for VTT-E (nearly 100 times), both of which are significantly decreased compared with the skin virulence of the original VG9 and VTT strains [4].

**Table 1:** Skin virulence of VG9-E and VTT-E in rabbits.

Dose	Swollen diameter (VTT-E, cm)			Swollen diameter (VG9-E, cm)		
(PFU)	3^th^ day	4^th^ day	5^th^ day	3^th^ day	4^th^ day	5^th^ day
10^7.06^	0.6	0.8	0.9	0.3	0.6	0.6
	red swelling	red swelling	red swelling	red	red swelling	red swelling
10^6.06^	0.4	0.6	0.8	0.1	0.1	0.2
	red swelling	red swelling	red swelling	a little red	a little red	red
10^5.06^	0.2	0.3	0.5	–	–	a dot
	a little red	red swelling	red swelling			
10^4.06^	a dot	0.1	0.2	–	–	–
		a little red	a little red			

### Immune Response against HIV-1 Env Induced by VG9-E and VTT-E

The humoral and cellular responses to HIV-1 Env for the heterologous (DNA/recombinant vaccinia viruses, rVV) prime-boost strategy were evaluated to explore the potential of using VG9 as a vaccine vector. The anti-Env binding antibodies in mice immunized with VG9-E (*n* = 10) and VTT-E (*n* = 10) were detected with an ELISA kit. All serum samples were diluted 10 times and tested. Three of the ten sera were positive in the VG9-E group and four were positive in VTT-E group. Although the positive values for the binding antibody induced by VG9-E were higher than those induced by VTT-E, the positive ratio between the two groups was not significant (*P*>0.05, data not shown), suggesting that VG9-E could induce effective binding antibodies against HIV-1 Env in inoculated mice, the same as VTT-E.

**Figure 3:**
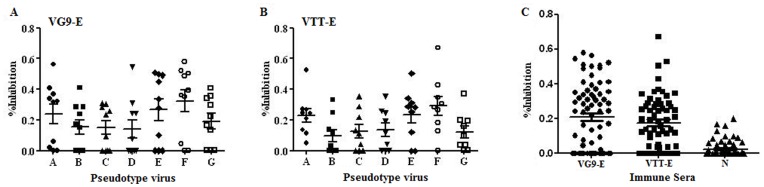
Neutralizing antibody assays. All serum samples were collected two weeks after the last rVV inoculation, then were inactivated and diluted 30 times before tested. The pseudotype neutralizing inhibition ratios of the sera induced by DNA/VG9-E (A) and DNA/VTT-E (B) immunization. The x-axis indicated different pseudoviruses, A-G respectively represented S19-15, 2–6, S21-28, 11058, 11308, MS208, and N192D. C: Inhibition of VG9-E and VTT-E vaccinated sere against all seven pseudotype viruses. The data of negative control was got from mice inoculated by only a single dose of VG9-E or VTT-E.

The neutralizing antibody induced by the two recombinant viruses was evaluated using a pseudovirus-based assay incorporating seven strains of pseudoviruses. All serum samples were diluted 30 times and tested. Our data showed that virus neutralization for sera from the VG9-E against seven pseudoviruses was F > E > A > G > B > C >  = D, with A, E, F, and G showing a statistical significance compared with B, C, and D (*P*<0.05, Figure 3A). Virus neutralization results for sera from the VTT-E group indicated F > E  =  A > D  =  C  =  G > B, with A, E, and F showing a statistical significance compared with B, C, D, and G (*P*<0.05, Figure 3B). For the same pseudovirus, we could not determine any statistical significance between the sera from the VG9-E and VTT-E groups. Although the positive ratio for both groups was different, the neutralizing capacities of antibodies induced by VG9-E and VTT-E against pseudotype viruses were not statistically significant (*P*>0.05, Figure 3C). Therefore, the ability to induce humoral response against the HIV-1 Env antigen in mice by VG9-E was the same as VTT-E.

**Figure 4:**
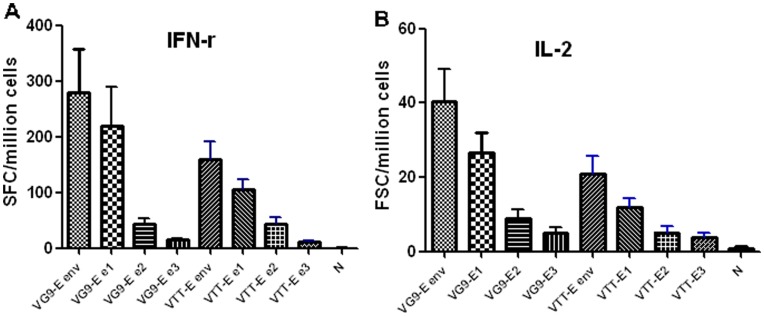
Env-specific splenic T lymphocytes secreted IFN-γ (A) and IL-2 (B) induced by VG9-E and VTT-E using ELISpot assay. The splenocytes were harvested and treated, then were added to 96 wells (in duplicate) at a seeding concentration of 4×10^5^ cells/well. HIV-1 PTE (Env) peptides were combined with three peptide libraries (e1, e2, e3) and added to the cells at a final concentration of 5 µg/mL for each peptide library. Data for all SFCs was subtracted from the background (mean SFCs without PTE stimulation). SFCs for each mouse were determined as the SFCs stimulated by all 480 peptides of PTE. The data of negative control was got from mice inoculated by only a single dose of VG9-E or VTT-E.

The cellular responses to HIV-1 Env for the heterologous (DNA/rVV) prime-boost strategy were evaluated by IFN-r and IL-2 ELISpot assay. The number of splenic T lymphocytes in the VG9-E group secreting IFN-γ and IL-2 stimulated by PTE (HIV-1 potential T cell epitope Env peptides) 1, 2, 3 (e1, e2, e3) differed. Significant statistical differences between the number of lymphocytes secreting IFN-γ and IL-2 are shown in Figure 4. The density of splenic T lymphocytes secreting IFN-γ, induced by VG9-E and VTT-E, was 279±78 and 159±32 SFCs per million cells (Fig. 4A), which was significantly different (t = 2.932, *P*<0.01). The proportion (SFC ≥50) of IFN-γ-secreting T lymphocytes induced by VG9-E and VTT-E was 91% (10/11) and 100% (10/10), respectively. The number of T lymphocytes secreting IL-2 induced by VG9-E and VTT-E was 40±9 and 21±5 SFCs per million cells (Fig. 4B), without a significant difference (t = 1.895, *P* = 0.0734, Group t test). The proportion of these positive cells (SFC ≥50) induced by VG9-E and VTT-E was 36% (4/11) and 0% (0/10), respectively. The cellular responses to HIV-1 Env of the two strains in the absence of DNA priming were similar and very low (not shown).

## Discussion

The VG9-E and VTT-E viruses containing the HIV-1 Env region were developed using standard homologous recombination techniques. No obvious differences in host cell range and diffusion were observed between the two recombinant viruses. This likely explains why the cell susceptibility of VG9-E and VTT-E remained unchanged.

This study indicated that the neurovirulence of VG9-E and VTT-E was decreased by more than 1000-fold when compared with their parent strains (VG9 and VTT) [4]. Therefore it suggested that the virulence of the two recombinant vaccinia viruses are highly attenuated *in vivo* compared to their parent virus. Moreover, we also validated the fact VG9-E virulence is weaker than that for VTT-E. We supposed that vaccinia virus VG9 is safer than its parent strain, VTT, not only as a vaccinia virus vector for HIVs, but also may as a vector for gene therapy of tumors.

After two inoculations with pDRVISV1.0-*env*, and one booster immunization with VG9-E or VTT-E, a low level of binding and neutralizing antibodies against the HIV-1 Env antigen were induced without any statistically significant difference between them. With respect to the cellular response, the proportion of T-lymphocytes secreting IFN-γ, induced by the two recombinant vaccinia virus strains, were dominant (nearly 90%) and the proportion of T cells secreting IL-2 was low (almost 10%). The cellular immune responses induced by VG9-E were stronger than those observed for VTT-E. It has been reported that virus strains with lower levels of virulence might induce stronger immune responses against foreign proteins than a virus with higher virulence [12]–[14]. This supports the fact that VG9-E is able to induce a stronger immunological response.

In this work, we have shown that VG9-E is highly attenuated *in vivo* and its virulence compared with VTT-E, was lower. VG9-E induced similar humoral immune response as VTT-E. However, the cellular immune response induced by VG9-E was higher than that did by VTT-E. Thus, VG9 may be an ideal replicating vaccinia vector for HIV vaccine research and development.

## Methods

### Materials

Primary chicken embryo fibroblasts (CEFs) were from 9- to 11-day-old specific pathogen-free (SPF) embryos; and cell lines included Vero, PK (15), CHO-K1, C6 and 293FT, and vaccinia virus VG9 and VTT strains were from our laboratory. A pseudovirus panel that contained 7 pseudoviruses (2–6, N192D, S19-15 and S21-28, 07_BC subtype; 11058, B subtype; 11308, C subtype; MS208.A1, A subtype), was used to determine the breadth and potency of neutralizing antibody induced by vaccinia vaccines. Pseudoviruses (11058, 11308 and MS208.A1) were provided by the NIH AIDS Research & Reference Reagent Program (ARRRP) [15], [16], and the other four viruses were constructed in our laboratory (S19-15, S21-28, 2–6, N192D) [17]. All HIV-1 Env pseudoviruses were produced and titered as previously described [18]. HIV-1 potential T cell epitope (PTE) Env peptides, designed to permit expression of the most frequent potential T cell epitopes embedded in the sequences of circulating HIV-1 strains of HIV-1 worldwide were also provided by ARRRP [19], [20], which were used to evaluate the magnitude and breath of the T-cell response induced by the vaccinia based AIDS vaccines [21].

### Construction of Vaccinia Virus Transfer Vector pJSC1175-*env* and DNA Vaccine pDRVISV1.0-*env*

The recombinant plasmid pJSC1175-*env* was constructed using a previously reported method [22]. Briefly, HIV-1 *env* was amplified from pcDNA3.1-ENV [23] using primers 1 and 2 (Table 2), and purified. The purified fragments and pJSC1175 [24] were digested with *Bam*HI and *Smal*I respectively. Plasmid pJSC1175 and *env* were ligated at 16°C overnight, resulting in the transfer vector pJSC1175-*env*. The strategy for constructing the DNA vaccine, pDRVISV1.0-*env*, which included the same HIV-1 *env* as VG9-E was similar to that outlined for pJSC1175-*env*, except that the vaccine vector pDRVISV1.0 [25], [26] was used as a backbone. The restriction endonucleases used were *Sal*I and *Eco*RV; the primers used were primers 5 and 6 (Table 2).

**Table 2:** Oligonucleotide primer sequences used in this study with restriction endonuclease sites underlined.

Primer	Sequence 5′−3′	Endonuclease
Primer 1	CGG GAT CCA TGA GAG TGA TGG GGA TCA GG	*Bam*HI
Primer 2	TCC CCC GGG TTA TTG CAA AGC TGC TTC AA	*Sma*lI
Primer 3	GCA CGG TAA GGA AGT AGA ATC AT	
Primer 4	TAC GCT CAC AGA ATT CGA GCT CG	
Primer 5	ACGCGTCGACGCCACCATGAGAGTGATGGGGATCAGG	*Sal*I
Primer 6	TGC AGG ATA TCT TAT TGC AAA GCT GCT TCA A	*Eco*RV

Primers 1 and 2 were used to amplify HIV-1 *env* gene which was used to construct transfer vector pJSC1175-env from pcDNA3.1-ENV. Primers 3 and 4 were used for the identification of two recombinant vaccinia viruses. Primers 5 and 6 were used to amplify HIV-1 *env* gene which was used to construct the DNA vaccine pDRVISV1.0-env from pcDNA3.1-ENV.

### Engineering and Screening of VG9-E and VTT-E

VG9-E and VTT-E were constructed using standard homologous recombination techniques. Recombinant VG9-E was obtained by infecting CEFs with VG9 at a multiplicity of infection (MOI) of 0.01, then cells were transfected with pJSC1175-*env* using Lipofectamine 2000 (Invitrogen, Carlsbad, CA). Recombinant VTT-E was constructed using the same strategy. The recombinant viruses were purified for more than six rounds. The two recombinant virus strains were propagated in CEFs and titered in Vero cells.

### Western Blot

Western blotting was used to confirm the expression of target protein. First, CEFs were infected with VG9, VTT, VG9-E, or VTT-E at an MOI of 10. Cells were collected after a 48 h culture period, lysed, and subjected to sodium dodecyl sulfate-polyacrylamide gel electrophoresis (SDS–PAGE). The cell lysates were used to determine the expression of ENV [27]–[29]. A diluted (1∶50) HIV-1-positive human serum sample was used as the primary antibody, and diluted (1∶1500) horseradish peroxidase (HRP)-linked goat anti-human immunoglobulin G (IgG; Zhongshan Goldbridge Biotechnology Co., Ltd, Beijing, China) was used as the secondary antibody.

### Immunofluorescence

Immunofluorescence methods [30]–[32] were used to determine the expression of HIV-1 ENV from VG9-E and VTT-E. Briefly, CEFs were grown to 90% confluence, then infected with 100 plaque-forming units (PFUs) of VG9-E and VTT-E. After adsorption for 90 min, cells were washed with culture medium and maintenance medium replenished. Cultures were incubated at 37°C/5% CO_2_ for an additional 48 h; then the supernatant was aspirated, cells were washed with phosphate-buffered saline (PBS) and fixed with 80% cold acetone. Human HIV-1-positive serum was added and fixed cells incubated at 37°C for 60 min. Cells were washed with PBS, followed by the addition of anti-human IgG conjugated to fluorescein isothiocyanate (FITC) and incubation in the dark at room temperature for 30 min. After washing with PBS, HIV-1 ENV was detected with the aid of a fluorescence microscope. Normal human serum, VG9 and VTT were used as negative controls.

### Viral Replication and Immunostaining of Infected Cells

CEFs in maintenance medium (culture medium containing 3% FBS) were infected with viruses at an MOI of 0.05. After a 90 min of incubation at 37°C, cells were washed three times with medium and replenished with fresh culture medium. Viral supernatant and infected cells were harvested at 72 h post-infection (p.i.). After three cycles of freeze–thawing, harvested samples were titrated in duplicate in Vero cells [33].

To determine host cell range and replication of the two recombinant viruses, six cell types were infected with the two recombinant viruses in maintenance medium [1], [34], [35] and viral plaques were detected after immunostaining [8]. Target cells were grown to 90% confluence, then infected with 100 PFU of VG9-E and VTT-E. Following adsorption for 90 min, cells were washed three times with culture medium and then incubated at 37°C for an additional 24, 48 or 72 h. A rabbit antibody against VTT was added and incubated for 2 h. Anti-rabbit IgG conjugated to HRP (Zhongshan Goldbridg Biotechnology Co., Ltd, Beijing, China) was added and incubated for 60 min to detect bound rabbit antibodies.

### Virulence Assay

Groups of female BALB/c and 4-day-old BALB/c mice (*n* ≥5 per group) were inoculated intracranially with serial 10-fold dilutions of VG9-E and VTT-E (10^2^ to 10^6^ in 30 µL or 20 µL of sterile PBS) to determine the 50% intracerebral lethal dose (ICLD_50_). Animals were observed daily over 14 days. The ICLD_50_ value was determined from mice that succumbed between 1 and 14 days p.i. by calculating the 50% end point using the method of Reed-Muench [9].

For skin virulence in rabbits, four dilutions (10^4^, 10^5^, 10^6^and 10^7^ PFU in 0.1 mL sterile PBS) of VG9-E and VTT-E and 0.1 mL sterile PBS (negative control) were injected intradermally on one side of each animal’s dorsal spine. The diameters of red swelling and necrosis sizes were measured and the difference between the two viruses was compared.

### Immunization Schedule for Immunogenicity Evaluation

Each mouse was inoculated with 50 µg of purified pDRVISV1.0-*env* suspended in 100 µL of PBS by the intramuscular (IM) route and subjected to electroporation (60 V, six times, once per second) at weeks 0 and 2. At week 6, following the second immunization with HIV-1 DNA plasmids, the mice were boosted with a single intradermal injection of VG9-E or VTT-E encoding HIV-1 proteins with a virus titer of 5×10^6^ PFU (suspended in 100 µL of PBS) at each animal’s dorsal spine. At the same time, only a single VG9-E and VTT-E (each for 5 mice) was inoculated by intradermal injection respectively as control. The mice were sacrificed 2 weeks after the last inoculation [36]–[40].

### Anti-Env Binding Antibody and Neutralizing Antibody

The anti-Env binding antibody levels were measured using an enzyme-linked immunosorbent assay (ELISA) kit for the detection of anti-HIV antibodies (Shanghai Kehua Bio-engineering Co., Ltd, Shanghai, China). Neutralizing antibodies were measured using a pseudovirus-based neutralization assay [18], [41]–[48]. Pseudotyped virus neutralizing inhibition ratio was calculated as follow: inhibition ratio  =  [1 − (value of the tested sample − value of cell background)/(value of virus control − value of cell background)] ×100%.

### ELISpot Assays

The splenocytes were harvested and treated according to the previous reports [37], [39], [40]. Splenocytes were added to wells (in duplicate) at a seeding concentration of 2 or 5×10^5^ cells/well. HIV-1 PTE Env peptides were combined with three peptide libraries and added to the cells at a final concentration of 5 µg/mL for each peptide library. The positive control was stimulated with PMA (50 ng/mL) and ionomycin (1 µg/mL), and the negative control lacked stimulation with any peptide. The splenocytes secreting IFN-γ and IL-2 were detected using ELISpot kits (BD Biosciences). The resulting spots were counted with an Immunospot Reader (CTL, Cleveland, OH). the number of specific IFN-g or IL-2-secreting T cells was calculated by subtracting the negative control value from the established SFC counts and only values >4-fold of negative control and not less than 50 SFC per million cells were considered as positive [49].

### Statistics

Statistical analysis was performed with the Statistical Analysis software application (version 2004; ISHIDA) in consultation with a biostatistician. Unpaired, two-tailed *t*-tests were used to assess significance with *p*-values less than 0.05 considered statistically significant.
